# O.1.1-3 Measuring the mental health benefits of community-based physical activity programmes-what are the most important indicators for pragmatic evaluation?

**DOI:** 10.1093/eurpub/ckad133.079

**Published:** 2023-09-11

**Authors:** McGrath Aisling, Niamh Murphy, Matthews Evan, Wagemakers Annemarie, Tully Mark, Verkooijen Kirsten

**Affiliations:** South East Technological University, Ireland; South East Technological University, Ireland; South East Technological University, Ireland; Wageningen University; University of Ulster; aisling.mcgrath@setu.ie; Wageningen University

## Abstract

**Purpose:**

While health enhancing physical activity (HEPA) initiatives can improve mental health, there is a lack of standardised measures to evaluate mental health outcomes. The need for the current work emerged from discussions amongst researcher and practitioner members of the HEPA promotion in socially disadvantaged groups Working Group of the HEPA Europe network. This study aimed to identify the most relevant indicators of mental health and well-being in community-based HEPA initiatives in Europe, and determine optimal assessment methods.

**Methods:**

An adapted, two round, Delphi method (guided by an indicator framework to categorise mental health indicators based on the dual-continua and socio-ecological models) was conducted with 20 experts in the field of mental health and physical activity from Denmark, the Netherlands, the UK, and Ireland. Experts selected the most important indicators and agreed consensus on definitions and their application, where consensus ≥50% signified important indicators.

**Results:**

Experts compiled 66 (n = 21 outcome, n = 45 determinant) indicators. Top rated indicators for the evaluation of HEPA initiatives were self-rated mental health (69.2%), physical activity (69.2%) life satisfaction (53.8%), stress (53.8%), loneliness (53.8%), social participation, network, connection and support (53.8%). Consensus on definition and application of the nine indicators varied (44.4% - 100%), with no consensus on a standardised measurement tool reached.

**Conclusions:**

While this study highlights a lack of conformity for evaluating mental health and wellbeing outcomes, it suggests utility in an agreed definition and application of nine indicators for the evaluation of HEPA initiatives, with social determinants of particularly high importance across all countries. Suggested measures are provided to aid community based practitioners measure mental health outcomes of physical activity programmes. Further research is recommended to develop a standardised measurement tool that can be utilised across other European countries and its implementation tested.

